# Experimental and Numerical Characterization of Electrospun Piezoelectric Polyvinylidene Fluoride Nanocomposites Reinforced with Silver Nanoparticles

**DOI:** 10.3390/ma18071467

**Published:** 2025-03-26

**Authors:** Strahinja Milenković, Fatima Živić, Nenad Grujović, Katarina Virijević, Aleksandar Bodić, Danilo Petrović

**Affiliations:** 1 Institute for Information Technologies, University of Kragujevac, 34000 Kragujevac, Serbia; katarina.virijevic@uni.kg.ac.rs; 2 Faculty of Engineering, University of Kragujevac, 34000 Kragujevac, Serbia; zivic@kg.ac.rs (F.Ž.); gruja@kg.ac.rs (N.G.); abodic@uni.kg.ac.rs (A.B.); petrovicdanilo1999@gmail.com (D.P.)

**Keywords:** PVDF/AgNP composite nanofibers, silver nanoparticles (AgNPs), piezoelectricity, electrospinning, numerical modelling

## Abstract

This study focuses on preparing piezoelectric polyvinylidene fluoride (PVDF) nanocomposites reinforced with silver nanoparticles (AgNPs) using an electrospinning process. The aim of this study is to assess AgNPs’ influence on the piezoelectric properties of PVDF and, therefore, create an optimal piezoelectric composite with enhanced properties, enabling its application in various fields both as sensor and actuator. Because electrical stimuli have proven to have a positive influence in tissue engineering, combined with AgNPs, which have antimicrobial properties, these composites demonstrate a promising opportunity for application as biomedical scaffolds. The proposed scaffolds were characterized by scanning electron microscopy, energy-dispersive X-ray spectroscopy and Fourier transform infrared spectroscopy. In addition, mechanical properties are studied through tensile tests, while piezoelectric response is measured on an in-house built setup coupling mechanical stimuli and electrical response monitoring. An experimental test was combined with numerical simulations through the COMSOL Multiphysics version 6.3 software package, and this paper also presents a short review of the numerical and analytical methods used for the modelling and simulation of piezoelectric composites.

## 1. Introduction

Poly(vinylidene fluoride) (PVDF), a piezoelectric polymer, has been extensively studied for usage in sensor and actuation applications [1,2]. Its piezoelectric properties make it particularly suitable for converting mechanical stress and strain into electrical energy, making it valuable for various energy harvesting applications [3,4]. In recent years, research has focused on polar polymers that exhibit piezoelectric, pyroelectric and ferroelectric properties, including PVDF, copolymers of trifluoroethylene and vinylidene fluoride and, as well as aromatic properties, including polyurethanes, vinyl cyanide and acetate, and nylons [5]. PVDF can exist in multiple crystalline phases (α, β, γ, δ and ε), but only the β phase possesses piezoelectric, pyroelectric and ferroelectric characteristics [6,7,8,9] suitable for energy harvesting applications.

In order to fabricate PVDF with a high β-phase fraction, certain challenges need to be addressed. The most common method to enhance the β-phase fraction in PVDF is mechanical stretching, which promotes transformation from the α phase [8,10,11]. This transition is also highly affected by the temperature and stretching ratio, which, in turn, impact the degree of crystallinity and microstructure, ultimately influencing the macroscopic properties of said material. PVDF’s fabrication, polymerization and process parameters can also affect the formation of certain phase contents [12]. Various fabrication methods have been investigated, including electrospinning, solution casting, spin coating, hot pressing, self-poling, melt blending, soft lithography and additive manufacturing techniques such as 3D printing and solvent evaporation-assisted 3D printing [2,4,13,14].

Electrospinning, as a fabrication method, employs mechanical stretching of a liquid solution droplet under a high electric field to produce long nanofibers [15,16]. Both the stretching process and the applied electric field facilitate dipole alignment in PVDF, leading to a higher β-phase fraction in the resulting nanofiber mats [17,18,19].

Adding fillers into the PVDF matrix is proven to increase electroactive phase content in PVDF, as well as mechanical properties. This fillers include barium-titanate (BaTiO_3_) [20], Graphene Oxide (GO) [21], silver nanoparticles (AgNPs) [22,23,24], zinc oxide (ZnO) 25], etc. In addition, silver nitrate (AgNO_3_) is a well-known antimicrobial agent that inhibits the growth of microbes; it has proven antimicrobial and anti-cancer effects due to its release of silver ions (Ag+) that disrupt the membranes of cancer cells and microorganisms [2].

Ag’s proven cytotoxic effect on cancer cells combined with PVDF piezoelectric properties paves the way for the development of smart, multifunctional materials for biomedical applications such as cancer treatment [2]; tissue engineering for orthopedics [25,26] and wound healing [27]; and biomedical sensors [28]. In our previously published paper [2], we proved that PVDF/AgNPs nanomats show both a prominent effect on the MDA-MB-231 breast cancer cell line and non-toxicity on the healthy MRC-5 cell line, as well as antibacterial activity against *S. aureus* and *P. aeruginosa* at Ag contents of 0.3% Ag, which was one of the reasons why we choose to test PVDF + 0.3% AgNPs in this work.

Fourier transform infrared spectroscopy (FTIR) is an effective method for the characterization of electroactive phases in PVDF [2,12,29]. In addition, Raman spectroscopy [12] and X-ray diffraction [10] are also utilized for the same purposes.

In order to measure piezoelectric response, various methods are reported in the literature since there are no established experimental methods for measuring all piezoelectric properties of a material. Piezoelectric Force Microscopy (PFM) is based on deformation induced by voltage on piezoelectric structures; this method can be compared to Atomic Force Microscopy (AFM) [30]. Generally, piezoelectric properties can be determined using commercially available equipment, but many researchers build their own in-house equipment, such as impedance analyzers [31], or run simulation measurements of mechanical load and electrical voltage [32] or mechanical strain and voltage in quasi-static conditions [33] or under frequent load conditions [21,23,34]. In addition, some authors reported testing piezoelectric nanomats under stochastic test conditions simulating those found in nature [20].

Modelling and simulating piezoelectric composites’ piezoelectric response is currently cutting-edge research [20,32,35] focused on establishing methods for easier design and the prediction of material behaviour to tailor it for specific purposes. A short review of numerical and analytical methods will be presented in the next chapter.

In this study, the linear electroelastic behaviour of piezoelectric composites was investigated by applying FEM modelling, where previously experimentally obtained material properties are utilized. For this purpose, COMSOL Multiphysics version 6.3 software was used. 

## 2. Numerical and Analytical Modelling of Piezoelectric Composites

Computational models of piezoelectric composites have been developed and studied to support different aspects of real case applications, as previously listed [36,37,38,39,40]. Each of the specific applications focuses on some of the available properties of the piezoelectric material, such as maximizing the output voltage or power. Hence, it is necessary to optimize the composite structure and performance in order to provide necessary material property for a specific application. Experimental procedures for adjusting the composite properties are tedious and long-lasting. These experiments often lack precise predictability, particularly in the absence of the physical analytical laws for the complex material structures. As a result, numerous repetitions of the experimental samples and corresponding tests are typically required. Computational models and related simulations of the material performance under various conditions have immensely shortened the time for material discoveries. They also provide more comprehensive understanding of the associated phenomena in material behaviour, thus justifying efforts engaged in developing new material models and new methods in simulating their behaviour. This is extremely important especially for conditions that cannot be easily set up in a real lab environment, such as mimicking human tissue environments. However, the complexity of possible piezoelectric composite structures, together with simultaneous coupling of different influential conditions, represents a significant challenge in the development of the computational models.

Theoretical analytical and mathematical models, very often physics-based models, are the foundation for the development of modern computational modelling and simulations, including AI-based predictions for material behaviour. Some of the first theoretical frameworks for the prediction of properties of piezoelectric composites with different reinforcements were reviewed in [36], together with the introduction of connectivity theory by Newnham [41], where different standard types of connections between the reinforcement and matrix were suggested, to distinguish different types of piezoelectric composites where 0-3, 1-3, 3-3 and 2-2 composites are amongst the most utilized ones. These defined connectivity patterns further enable the definition of series and parallel models that represent connections between piezoelectric composite layers or reinforcing fibres and particles, thus enabling well-defined approximations in numerical models. In the case of PVDF-based composites, 0-3, 1-3, 3-3 and 2-2 connectivity patterns are of interest. The coupled behaviour present in piezoelectric composites, originating from mechanical and electrical influences, has been a subject of many works, also by introducing specific boundary conditions.

In general, micromechanics models were first used for the modelling of piezoelectric materials, and they are still the most significant theoretical models [36]. The four most prominent micromechanics models for piezoelectric composites are as follows [36,40]:

1. The dilute approximation;
2. The self-consistent method;
3. The Mori–Tanaka mean field method;
4. The differential scheme.

More detailed categorisation of the existing material models used in the modelling of piezoelectric behaviour are given in [40]. This very recent review presented the latest developments of the numerical methods for piezoelectric composites, including homogenization methods [40].

Modelling of the piezoelectric composites, according to the specific approach, can be categorized as follows [40]:

5. Analytical models:
  - Micromechanics models based on averaging material properties through the constituent phases and with different types of connectivity, where 0-3 represents the random dispersion of elements within the composite that have isotropic properties; 1-3 represents structures with fibres where one observed phase can be connected through series (meaning constant properties within the composite) or parallel connections (where weights are calculated); and 3-3 connectivity represents the 3D distribution of elements, but, unlike the 0-3 type, it has a somewhat arranged orientation and alignment of the phases. However, these models cannot capture localized behaviour within small domains due to the averaged properties, such as commonly appearing fluctuations in properties (both mechanical and electrical) within the small domains in different phases and along their interfaces.
  - Models based on Eshelby equations:
    i Dilute approximation models;
    ii Mori–Tanaka-based models;
    iii Extended Mori–Tanaka models, such as Mori–Tanaka–Eshelby models;
    iv Self-consistent models and their extended models;
    v Extended rule of mixture models.
  - Models based on asymptotic homogenization (or periodic homogenization).
6. Numerical models or Finite Element (FE) models, which use representative volume elements (RVEs) throughout the 3D volumetric space of the composite for the calculation of composite properties and responses to external influences, where the definition of the size and shape of RVEs essentially determines the model validity. FEMs can capture localized behaviour to some degree, depending on the defined RVEs, with several established approaches, also related to the computational resources needed for calculations:
  - Linear FE models (usually for elastic behaviour or small deformations);
  - Nonlinear FE models (large deformations, elasto-plastic material or nonlinear contacts);
  - The representative volume element method;
  - The theory of periodic boundary conditions;
  - Calculation methods for the effective coefficients;
  - Optimization of piezoelectric behaviour with different possible methods, such as the Discrete Material Optimization (DMO) method.

However, the accuracy of each model is closely related to the defined boundary conditions that, on the other hand, strongly depend on the observed specific application case. The developed micromechanics models are also used for the calculations of the effective composite properties, such as the effective elasticity modulus, effective piezoelectric coupling coefficients and dielectric coefficients. Each of the currently existing models have certain limitations for use. For example, the self-consistent method is not suitable for composites where there is a large difference in the moduli of the matrix and reinforcements. The number of reinforcements, or their volume fractions, have a very significant influence on the model accuracy. For example, the Mori–Tanaka and dilute methods are relevant methods for volume fractions lower than 20%.

The Mori–Tanaka method utilizes averaged stress and strain, strain concentrations in the reinforcements that are dependent on the plastic domain within the material microstructure, strain and load distribution in the plain, electrostatic field and the overall shape of the studied sample [42]. The dilute model assumes that there is a large space and no interactions between the reinforcement inclusions. Hence, it can be assumed that concentration tensors in reinforcements can be observed as independent of their volume fractions, which also means that this method is not suitable for high volume fractions. Determination of the concentration tensor fields can be performed in different ways, such as using Green’s function [43]. The differential method deals with nonlinear problems, thus requiring large amounts of computational resources for solving ordinary differential equations. When considering computational resources and explicit solutions for the effective material properties, the Mori–Tanaka and dilute methods stand out for their easy use and are very often used in material modelling in general, including piezoelectric composites [38,39,40].

A summary of the advantages and limitations of different modelling approaches is given in Table 1.

From a theoretical point of view, it is possible to design PVDF-based piezoelectric composites with different connectivity patterns, as defined by [41]. However, in real case applications, reinforcements in the form of continuous long unidirectional fibres, or as nano and micro particles and their hybrid structural forms, are among the most commonly used composites. They can be efficiently represented by 0-3, 1-3 and 2-2 types of connections within the two-phase, three-phase and multi-phase composites, including porous structures.

Other micromechanics models have also been developed and studied, including the very recent approach of using artificial intelligence (AI) for material simulations [44,45]. Different analytical solutions have been implemented in the existing micromechanics models, considering different boundary conditions, to achieve the coupling of the mechanical and electrical phenomena in piezoelectric materials and determine effective composite properties [36,46]. Rigorous boundary conditions are still subject to research [40].

Material models can be further used to predict material behaviour, such as the recent mathematical model for the prediction of the harvested energy by piezoelectric composites [47]. This model can enable the study of different compositions for the optimal material design to suit the intended application. The recent approach focused on damage mechanics through phase-field modelling what is valuable for designing flexible piezoelectronics for a range of applications [48]. These models are relevant for large deformations and can capture nonlinear behaviour, which is a profoundly important property of PDVF-based piezoelectric composites that exhibit large failure strains [49]. Phase-field modelling uses thermodynamic principles together with kinetics and required input data about the microstructure to be able to predict microstructural evolution during the functional piezoelectric behaviour. Phase-field models employ Allen–Cahn and Cahn–Hilliard equations to include the interfaces between the different phases with the composite microstructure [50,51].

There are continuous efforts to upgrade FEM methods, focusing on better efficiency with lower computational resources needed for data processing [52]. Two-dimensional discretization within a single layer is a common FEM approach, but it lacks sufficient accuracy in simulation piezoelectric behaviour, especially for thick composites. Hence, 3D models and new FE models have been developed, often using combinations of 2D methods for mechanical displacements with 3D layerwise-like approximation for the electric potential field that can be used for both thin and moderately thick composites [53].

In 2D models, the distribution of the electric potential through the thickness of the composite is usually assumed to be constant, which can result in larger differences between the model and experimental results in the case of electrospun PVDF-based nanocomposites. Very fine discretization of the thickness can capture nonlinear behaviour and, together with carefully considered electric boundary conditions, the model can be designed with a moderate number of field variables to not require excessive computing power [53]. Commonly assumed linear or constant electric potential fields through the composite thickness can result in numerical solutions (for mechanical displacement and electric potential) that do not converge to the same exact solution, even with a finer meshing [53].

Currently developed 3D FEM models of piezoelectric composites use 3D representative volume elements (RVEs), are suitable for modelling thick piezoelectric structures and, often, can provide the exact solutions through solving the constitutive equations in three-dimensional space under certain prescribed boundary conditions (both the mechanical and electrical boundary conditions) [54]. If focusing on the lower computational resources with retained accuracy, layerwise theory has been used for composites that exhibit laminar structures [53].

Some recent models have combined FEM modelling with extended Eshelby–Mori–Tanaka micromechanics models for determining effective composite properties [54,55]. This model suggests that large piezoelectric coefficients, with low values of mechanical elastic compliance and low dielectric permittivity, are the best combinations for achieving high energy conversion efficiency in the case of energy harvesting applications using flexible piezoelectric composites. Another approach in FEM modelling has utilized Hamilton’s variational principle in the specific cases of plates and beams [56]. Coupling of the electric potential with mechanical displacements represents a complex task in 3D FEM modelling and is still under investigation, whereas the definition of the appropriate RVEs has the most profound effects on the model accuracy. In the case of a polymer matrix reinforced with nanostructures such as nanotubes, molecular interactions play a detrimental role and traditional continuum mechanics cannot be used for modelling, with new approaches suggested through the introduction of an effective continuum fibre that can be further used in micromechanics models [57].

Recent approaches in the modelling of PVDF-based piezoelectric composites have considered flexoelectricity, or the coupling of the resulting changing strain gradients and associated electric potential fields [58]. However, when flexoelectricity is considered for anisotropic structures, the contribution of the shear components is still not well understood, since it is hard to determine it in experimental conditions and due to the simultaneous influences of the mechanical properties of the composite.

A micromechanical model has been developed for finding effective properties of hybrid orthotropic composites where a piezoelectric matrix made of polymer is reinforced with piezoelectric particles [39]. This model showed good accuracy for the low volume fractions of reinforcing particles. Anisotropy of PVDF fibre-based composites needs to be considered in material models with several approaches, such as considering specific equations for predicting effective dielectric properties using the Knott model [59], piezoelectric coefficients using the Furukawa model [60,61] , or using other theoretical models [39,62]. Some micromechanical models have considered anisotropy in composite structures and used different homogenization models, such as the models by Voigt [63], variational principles and the derivation of bounds by Hashin and Shtrikman [64], the Halpin–Tsai equations based on the self-consistent method [65,66], the upgraded self-consistent method-like incremental self-consistent (ISC) method [55], the Eshelby–Mori–Tanaka model [39,67], including several extended models [36,68,69,70]; and the asymptotic homogenization method for developing analytical equations [71]. FEM modelling has also been used for anisotropic structures [39,72,73,74]. In the case of high volume fractions of the reinforcements, the Eshelby–Mori–Tanaka model showed good accuracy [55].

Kuo and Huang [69] developed analytical equations for the electrostatic field based on the 3D anisotropic inclusion method and provided explicit electrostatic tensors in line with Eshelby tensors. They also developed analytical expressions for the composite effective properties that depend on the volume fraction and phase properties (e.g., reinforcements), such as the orientation angle and shape of the phase, since these showed the most prominent influence on the resulting effective properties [69]. Several recently developed analytical models are shown in [75]. Odegard [72] developed a constitutive model suitable for the different range of volume fractions, different polymer matrices and geometries of reinforcements. An analytical model for 1-3 composites with anisotropic properties was developed by Kar-Gupta and Venkatesh [73]. This model, including a method to determine 45 material constants for piezoelectric composites, showed good accuracy in longitudinal directions. Optimization of the composite design can be realized based on these analytical models [75].

It is challenging to establish a 3D FEM model for composite structures that can simulate the simultaneous influence of electric potential fluctuations and mechanical displacements in micro domains throughout the volume, especially considering the high computational costs of this method of processing data. The definition of RVEs that can represent hybrid non-uniform composite structures is also complex and determines the validity of the material model. A recent approach is to use artificial intelligence (AI) and new machine learning (ML) algorithms that are applicable for a range of thicknesses and compositions, including hybrid composites [76,77]. Data-driven ML material models enable so-called meshless modelling, usually based on the data generated through experiments. Further challenges associated with experimental datasets to be used in AI/ML processing is their diversity of formats and common lack of metadata, indicating the urgent need for standards in materials science related to data and metadata for AI/ML-ready datasets. This is usually overcome by using experimental data from trusted sources, where the most common method is to perform comprehensive experimental tests and create AI/ML-ready datasets in line with data science principles.

New ML algorithms have been developed focusing on reducing the time and computational costs and especially to serve in the optimization of material properties to provide desirable composite strength or strain [78], optimal composite patterns [79] or to address coupling influences and predict the properties of complex composite structures [80]. A decision tree classification ML model, complemented with regression models, can accurately predict various mechanical properties of fibre-based composites, such as the shear modulus or modulus in longitudinal and transverse directions [81]. Artificial neural network (ANN) models with 20 input and 11 output parameters have been used to predict the electromechanical properties of piezoelectric composites with 99.998% accuracy [82]. An ANN model was used to predict conductivity and strain in nanocomposites, reducing the 3.5 h time for FEM simulations to 0.25 s by using an ANN, with excellent accuracy [83]. ML methods have also been used in relation to fabrication technologies and subsequent correlations with resulting material properties [84], such as predicting the fibre diameter of PVDF depending on the electrospinning parameters [85], or to optimize fibre diameter according to the desired material responses [86].

A deep neural network can be used to approximate any function regardless of its physical meaning and, as such, it can replace commonly used numerical FEM-based methods or analytical material models, including for modelling material structures of hybrid piezoelectric nanocomposites [76]. Recent approaches in material modelling have used physics-informed neural networks (PINNs) through solving partial differential equations (PDEs) that govern the material physical system and where specific boundary conditions can be defined [87]. For material modelling through PINNs, it is important to properly define the loss function in relation to PDEs, including boundary conditions in relation to setting collocation points. Physics-informed neural networks can be used to create accurate models for known physical systems, including for FE and continuous domains within the material structure, and can deal with both elastic and inelastic boundary conditions. It is common to use some homogenization methods, such as analytical homogenization solutions that are again based on some finite blocks within the material structure.

RVEs used in FEM models are a relatively simple solution to represent the whole material structure, but they lack the ability to represent dynamic changes within micro and nano domains, meaning that local distribution that considers multiphysics is impossible to represent with RVEs. A new approach is the introduction of a repeating unit cell (RUC) that can be flexible over domains, including nano and micro domains within a composite structure that commonly exhibits non-uniform distributions of reinforcements [88]. The difference between RVEs and RUCs is that RVEs are applied for statistically homogenous structures with rigid boundary conditions for the whole structure, where micro domains are hard to interpret, while RUCs are small blocks of material volume within the arrays periodically appearing throughout the whole material volume that always consider microstructural features and their interdependences, and which exhibit flexible boundary conditions related to these periodic arrays. RUCs are used to build periodic arrays rather than the whole material volume, and these arrays can have different microstructural features, depending on the observed material structure. In the case of RUCs’ use, localized homogenization is applied to provide a set of properties such as moduli or local stress fields, instead of one global value for each property, where arrays are commonly approximated by square, hexagonal or rectangular shapes for nanocomposites. Recently introduced RUC-based modelling is suitable for AI data processing due to its stability, significantly lower time needed for processing and very quick convergence, which enables comprehensive parametric study and very fast finding of the optimal material model [88]. However, defining the boundary conditions in the case of RUCs is still very challenging.

A multiphysics deep homogenization neural network (MDHN) has been developed for transversely isotropic piezoelectric composites where RUCs were used with periodic boundary conditions [76]. Changes in the micro domains of electric potential and mechanical displacements are simultaneously considered through PDEs related to both the matrix and reinforcements and observed as separate material phases. Two different neural networks were used to solve PDEs to avoid an otherwise commonly applied artificial interphase layer that is usually introduced into ML algorithms to perform smooth transitions between the matrix and reinforcement phases. Sharp transitions of the stress at the interface of these two distinctly different composite phases was addressed through the loss function and trainable weights.

Very often, FEM-based virtual simulations are used to generate datasets for training AI/ML models in the absence of AI/ML-ready experimental data [79,82,89]. The combination of FEM with AI/ML technology has shown excellent results [82], including a new approach to incorporate an AI surrogate material model into the FE solver for automatic analysis and predictions [90,91,92]. However, in the case of fibre-reinforced piezoelectric composites, the use of ANNs is still under study. Discoveries in materials science propelled by the use of advanced AI and ML algorithms have gained significant attention, belonging to the novel research area of Materials Informatics that has started to emerge [93,94].

## 3. Materials and Methods

Poly(vinylidene fluoride) (PVDF, Mw~180.00 by GPC) was purchased from Sigma Aldrich (St. Louis, MO, USA). Acetone (Ac, ≥99.5%) was purchased from Honeywell (Charlotte, NC, USA), and dimethylformamide (DMF, ≥99.5%) and silver nitrate (AgNO_3_, ≥99.9%) were purchased from Fisher Chemical (Waltham, MA, USA). In this study, as well as in a previous one [2], all chemicals were applied without further purification.

### 3.1. Fabrication of PVDF Nanofibers with Incorporated AgNPs

For PVDF electrospinning, the earlier-determined [2], most conductive solution was prepared. Parameters are given in Table 2.

The 21% PVDF solution was stirred at 80 °C for 3 h to achieve a homogenous mixture. Different concentrations of AgNO_3_ (0%, 0.3%, and 3% *w*/*w*) were then added to the solution, followed by stirring for an additional 12 h. The mixture was subsequently dispersed using an ultrasonicator and allowed to cool at 28 °C. The solution turned grey, signifying the formation of AgNPs [2].

The prepared solutions were transferred into a 5 mL syringe fitted with an 18-gauge needle. Electrospinning was performed under a voltage of 30 kV, with a flow rate of 0.5 mL/h and a needle-to-collector distance of 15 cm. The process was conducted under controlled conditions of 45% humidity and a temperature of 30 °C. After electrospinning, the nanofibers were cautiously collected onto aluminum foil and stored in a dark, room-temperature environment to allow the residual solvent to evaporate fully.

### 3.2. SEM and EDS Analysis

The nanofiber mat morphology was examined with a scanning electron microscope (SEM) (FEI Scios2 Dual Beam System, Hillsboro, OR, USA) at the Institute of Nuclear Sciences Vinča, University of Belgrade, Serbia. Square samples measuring 2 cm by 2 cm were prepared and gold-coated for 30 s to facilitate SEM analysis. The gold-coated samples were then carefully placed into an SEM operated at 10 kV. Additionally, energy-dispersive spectroscopy (EDS) was employed for elemental analysis to confirm the presence of Ag-loaded nanofibers.

### 3.3. FTIR Spectroscopy Analysis

The presence of crystalline phases in the electrospun PVDF samples was analyzed using transmission infrared spectroscopy with a portable FTIR/FT-NIR spectrometer (Interspec 301-X, Toravere, Estonia). The measurements were conducted in the range of 400 cm^−1^ to 1600 cm^−1^, with a resolution of 4 cm^−1^.

### 3.4. Electromechanical Tests at Macro Scale

To measure the thickness of electrospun materials, Trimos Labconcept Premium (Renens, Switzerland), samples were measured ten times each, in order to determine average thickness. In addition, specimen tensile testing was performed using a mechanical uniaxial testing machine, Ametek Brookfield CT3 Texture Analyzer (Devon–Bervin, PA, USA), equipped with 500 N load cells. Testing was performed with a testing speed of 10 mm/min, following the literature [95,96], due to a lack of standards for tensile testing of such materials [97]. Before testing, samples were cut to 45 × 20 mm dimensions. Since electrospun specimens are thin and sensitive, a paper frame with a 10 × 10 mm opening was introduced in which specimens were inserted using Scotch tape. The structure formed in this way was then inserted into tensile testing machine grips, after which frame sides were removed using scissors, as shown in figure 9.0, which ensures that tensile testing is performed on the electrospun specimen only [96]. Three samples of every material type were tested to ensure repeatability, and the average values are given.

**Figure 1:**
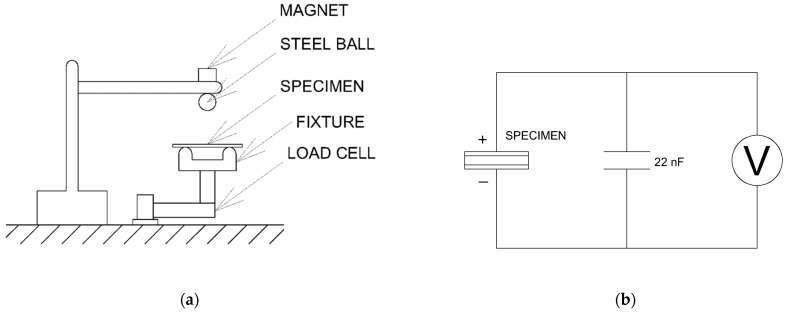
Piezoelectric testing fixture (**a**) and modified Sawyer–Tower circuit (**b**).

To determine the piezoelectric $d_{33}$ coefficient, a modified Sawyer–Tower circuit (Figure 1b) was employed [32]. This was coupled with load measurements through load cells, ensuring simultaneous load and electrical voltage measurements, as described in Figure 1a.

Testing was conducted by dropping a steel ball from a fixed height (50 mm) onto test specimens, measuring both load and electrical voltage [34] across a 22 nF capacitor; the voltage measured across the capacitor is later used to calculate the piezoelectric coefficient:

(1) $$d_{33}=\frac{Q}{F}=\frac{CV}{F}$$

where $d_{33}$ is the piezoelectric coefficient, *Q* is the charge in the capacitor, *F* is the applied load (measured), *C* is the capacitance of the capacitor (22 nF) and *V* is the voltage across the capacitor (measured). For voltage and load measurements, the HBM (Virum, Denmark) QuantumX A410 and CATMAN 3.4.1 Software were used. Three different samples were prepared for every nanocomposite configuration and examined, with the average value considered.

### 3.5. Finite Element Analysis (FEA)

#### 3.5.1. Piezoelectric Constitutive Relations

The direct piezoelectric effect can be expressed with the following equation [98,99]:

(2) $$P_{\mathrm{piezo}}=dT$$

where **P**_piezo_ represents the mechanically induced polarization, **d** represents piezoelectric charge constants matrix and **T** is the applied stress vector. The total electric displacement field **D**, including **P**_piezo_, is defined by the following equation [98]:

(3) $$D=\varepsilon^{T}E+dT$$

where **ε**^T^ represents the dielectric permittivity matrix under constant stress and E is the electric field vector.

The inverse piezoelectric effect can be expressed with the following equation [99]:

(4) $$S=d^{T}E$$

where S is elastic strain vector and **d**^T^ represents a piezoelectric charge constant matrix under constant stress. The elastic strain vector, including the inverse piezoelectric effect, can then be expressed as [98]

(5) $$S=s^{E}T+d^{T}E$$

where **s**^E^ represents compliance for the constant electric field. By combining Equations (3) and (5), the following equations are obtained [100]:

(6) $$\begin{array}{l} S=s^{E}T+d^{T}E\\ D=dT+\varepsilon^{T}E \end{array}$$

Previous equations represent the constitutive relations for linear piezoelectric materials in the strain–charge form. This equation can be expressed in other forms in which different coefficients are used. Accordingly, in addition to the strain–charge form, the stress–charge form is also often used, which is given by the following equations:

(7) $$\begin{array}{l} T=c^{E}S-e^{t}E\\ D=eS+\varepsilon^{S}E \end{array}$$

where **c**^E^ is the elastic stiffness coefficient matrix evaluated at a constant electric field, e is the piezoelectric stress coefficient matrix, et is the transpose of **e** and **ε**^S^ is the dielectric matrix evaluated at constant strain.

#### 3.5.2. FE Model of PVDF/AgNPs Nanocomposite

The 2D FE model of PVDF/AgNPs nanocomposites was created in COMSOL software version 6.3 in order to prove the concept and set the foundations for future research and calibration. Numerical analysis was performed within the electromagnetics–structure interaction module which couples linear-elastic statics with the constitutive relations of piezoelectric materials given in Section 3.5.1 and is used to investigate the behaviour of piezoelectric materials at the macro scale, capturing the global structural response without going into the microstructure of the material. In this paper, three configurations were considered: pure PVDF and two nanocomposite configurations—PVDF with 0.3% and 3% AgNPs. Silver particles were not physically modelled, but their influence was considered through material parameters.

FEM-based simulation material properties were determined from the experimental tests performed (details in Section 3.4) with the prepared piezoelectric samples and entered as input values to the COMSOL software version 6.3.

The piezoelectric coefficient $d_{33}$ was calculated based on the experimental tests using the modified Sawyer–Tower circuit based on the measured voltage across the capacitor connected to the sample (details in Section 3.4). In order to calculate the contact area between the steel ball and the specimen, the Hertzian contact theory was employed [101] to calculate its radius as

(8) $$a=\sqrt[{3}]{\frac{3}{8}FD{(k}_{1}+k_{2})}$$

where F is the measured load, D is the steel ball diameter and $k_{i}$ is calculated as

(9) $$k_{i}=\frac{1-\nu_{i}^{2}}{E_{i}}$$

where ν is Poisson’s ratio, E is the tensile modulus and subscripts i=1,2 denote the sphere and plate. The calculated contact diameter is imported as geometry in COMSOL software version 6.3.

The values of material parameter $d_{33}$ were measured for different variants of PVDF/AgNPs nanocomposites (0%, 0.3% and 0.5% AgNPs), and they were used in numerical simulations. Material relative partitivities were calculated from experimental data for equivalent capacitance of both the specimen and reference 22 nF capacitor. Other material characteristics, which were unable to be obtained from experimental work, were adopted from COMSOL’s material library and were kept the same for every nanocomposite configuration.

**Figure 2:**
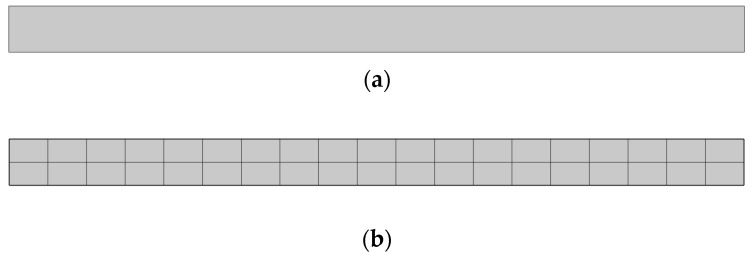
Geometry (**a**) and FE models (**b**) of PVDF and PVDF/AgNPs nanocomposite configurations.

The user-defined mapped quad mesh was generated with 38 elements. The geometry, which represents contact area radius and specimen thickness, and the FE model are given in Figure 2.

**Figure 3:**
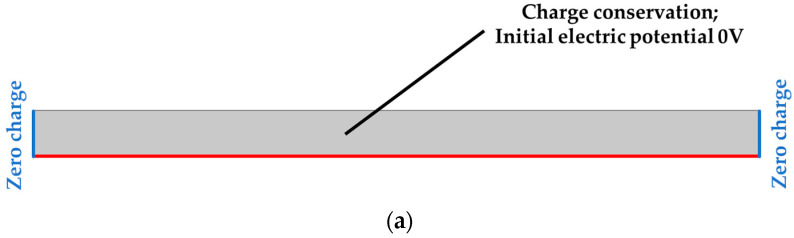

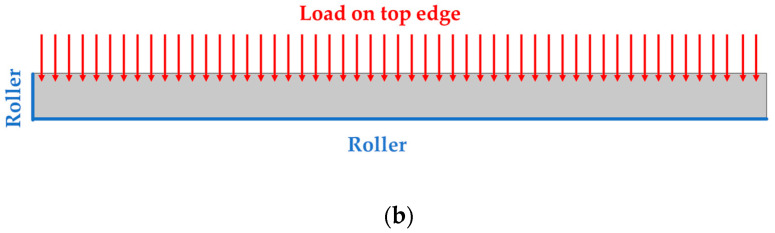
Loads and boundary conditions: electrical (**a**) and mechanical (**b**).

Boundary conditions (Figure 3) in the solid mechanics module were set as follows: the lower and left edge have an applied roller boundary condition, which limits displacement in the direction perpendicular to the boundary, while a compressive force of 1 N acts on the upper edge. The force value used within numerical simulations corresponds to the peak value of experimentaltesting results. In the electrostatics module, grounding is defined on the lower edge, which corresponds to the connection of the testing specimen to an electrode at ground potential, with zero charge on the side edges, while charge conservation and the initial value of the electric potential of 0 V are defined on all surfaces.

The processing hardware included 64 GB of RAM and an Intel Core i9-13900KF CPU running at 3.00 GHz (32 CPUs), while computational time was about 3–4 s. The results of the numerical analysis are given in the next chapter.

## 4. Results and Discussion

### 4.1. SEM and EDS Results

**Figure 4:**
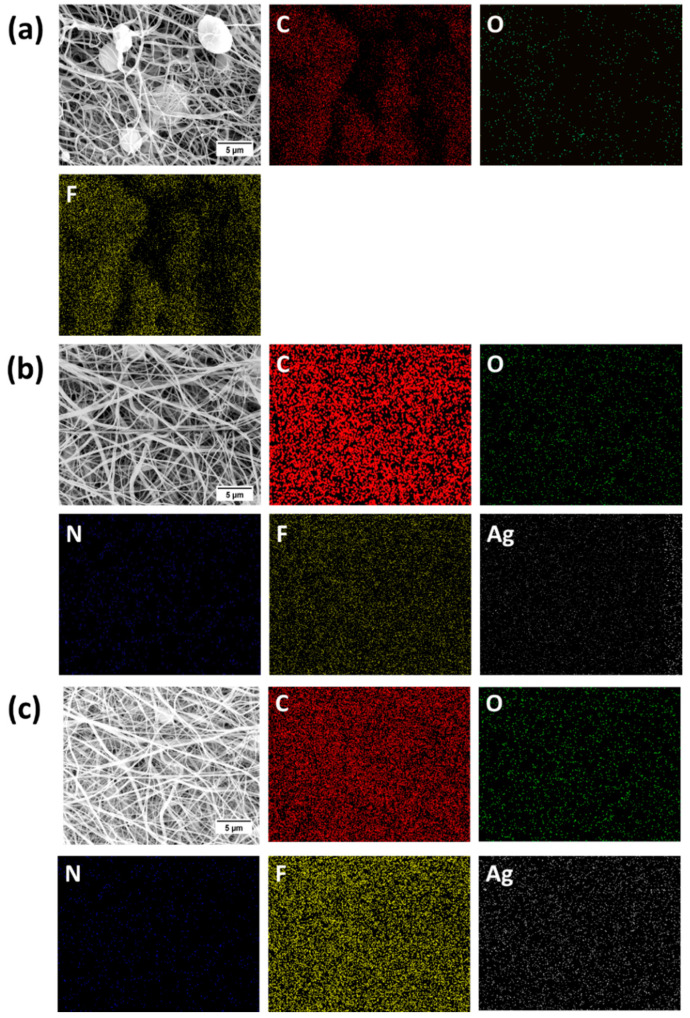
EDS analysis of (**a**) pure PVDF; (**b**) PVDF + 0.3% AgNPs; and (**c**) PVDF + 3% AgNPs nanofibers related to the distribution of C (red), N (dark blue), O (green), F (yellow) and Ag (white). The scale bar is 5 µm [2].

**Figure 5:**
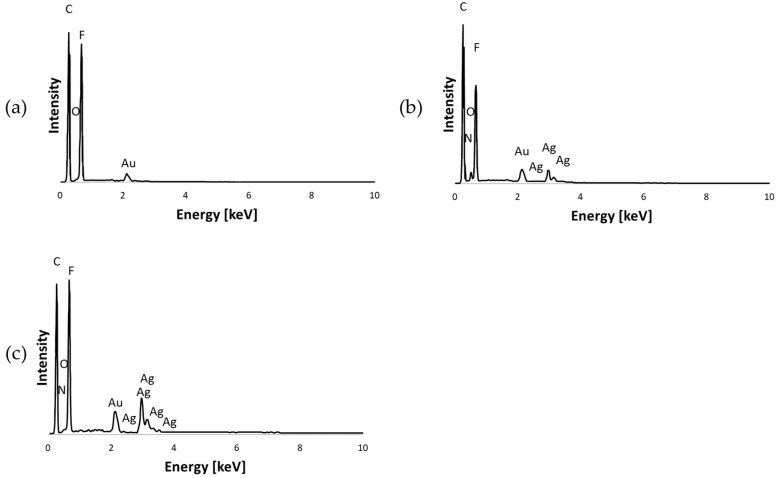
EDS diagrams of the elemental distribution of specimens: (**a**) pure PVDF; (**b**) PVDF + 0.3% AgNPs; (**c**) PVDF + 3% AgNPs.

SEM images of PVDF and PVDF/AgNP nanofiber mats are shown in Figure 4. The addition of silver nanoparticles to PVDF nanofibers shows significant differences in morphologies. However, due to a very low concentration of AgNO_3_, silver nanoparticles are not visible in SEM images. The addition of nanoparticles decreased the presence of beads which are notable in pure PVDF nanofiber mats. The reduction of beads is observed for both 0.3% AgNPs and 3% AgNPs specimens, although it is still present in structures. Also, Figure 5 shows EDS mapping of pure PVDF, PVDF + 0.3% AgNP and PVDF + 3% AgNP nanofiber mats. The existence of carbon (C), oxygen (O), nitrogen (N), fluor (F) and silver (Ag) atoms can be seen, confirming the successful integration of the silver nanoparticles in PVDF nanofibers. Additionally, Figure 5 shows the EDS spectrum, which confirms the existence and demonstrates the homogenous distribution of silver. 

The selection of AgNPs proportion in nanocomposites was based on our previous experimental work experience and literature review [23,24,102]. In our previously published study [2], we tested PVDF composites with AgNP concentrations of 0.1%, 0.3%, 0.5%, 1%, 3% and 5%. We concluded that higher concentrations (1%, 3% and 5%) show strong cytotoxicity against both healthy MRC-5 and MDA-MB-231 cancer cells. In addition, testing lower concentrations (0.1%, 0.3% and 0.5%) showed that 0.3% AgNPs have the greatest β-phase fraction and the best nanofiber morphology compared to the 0.1% and 0.5% concentrations in terms of the lowest bead formation and nanofiber diameters.

One of the main reasons for bead formation during the electrospinning process is low solution viscosity, leading to inadequate chain entanglement, causing the polymer jet to break up in droplets rather than forming continuous fibres. A higher solution concentration increases viscosity, which promotes the formation of bead-free fibres [103]. In addition, high surface tension can promote bead formation, so increasing the electrical field increases electrostatic forces that should overcome the surface tension and initiate jet [104]. Also, tip-to-collector distance can have an impact on the prevention of bead formation because complete solvent evaporation at optimal distance is what should be pursued [104]; however, this weakens the electric force, which can cause jet instability [103,105]. Further, insufficient charge density can be responsible for jet instability, hence promoting bead formation. Because of all of this, optimizing both the solution and process parameters is of peak importance. The addition of AgNPs to the PVDF solution can alter all of the beforementioned properties by increasing the solution conductivity [2,106], allowing jet stabilization, but only to some extent, as AgNPs can lead to aggregation, which increases surface tension, which again promotes bead formation.

### 4.2. FTIR Spectroscopy Analysis Results

**Figure 6:**
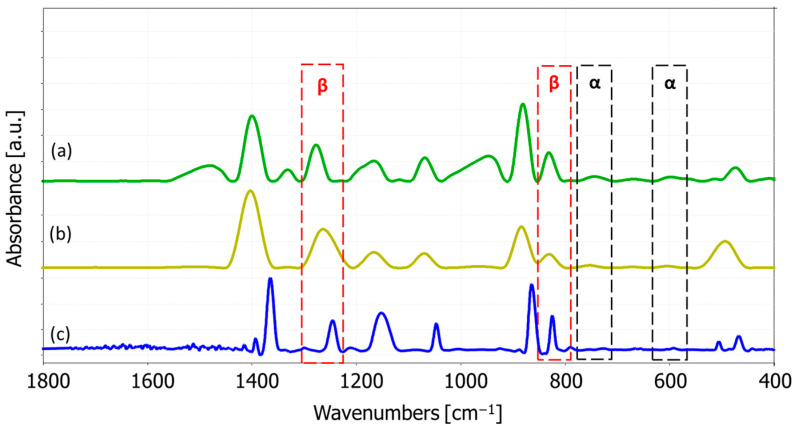
FTIR absorbance spectra of the specimens: (a) pure PVDF, (b) PVDF + 0.3% AgNPs, (c) PVDF + 3% AgNPs [2].

The FTIR spectra of pure PVDF and PVDF + AgNP nanofiber mats are shown in Figure 6. The absorption peak near 1400 cm^−1^ corresponds to the CH_2_ wagging vibration in PVDF. The band at 1180 cm^−1^ is associated with the asymmetric stretching vibration of the CF_2_ group, while the 1068 cm^−1^ band is attributed to the CH_2_ wagging mode [107,108]. The β-phase band at 874 cm^−1^ corresponds to CF_2_ symmetric stretching, while the α phase is identified by characteristic peaks at 763 cm^−1^ and 610 cm^−1^, related to CF_2_ bending. Peaks at 880 cm^−1^ and 841 cm^−1^ are linked to C-C-C and CF stretching vibrations of PVDF [107]. Unique peaks for the electroactive, piezoelectric β phase are observed around 445, 473, and 1275 cm^−1^ [29]. The chemical structure of the nanocomposite mat preserves the characteristic absorption peaks of pure PVDF. However, the absorption peak at 1662 cm^−1^, which indicates the presence of silver, was absent—likely due to the low concentration of AgNPs [2,109]. Nevertheless, the presence of silver was confirmed using EDS analysis.

Peaks at 840 cm^−1^ and 510 cm^−1^ provide specific insights into the phases of PVDF. When the 1275 cm^−1^ peak is present, but the 1234 cm^−1^ peak is absent, the 840 cm^−1^ and 510 cm^−1^ bands are attributed to the β phase. Conversely, if the 1275 cm^−1^ peak is absent while the 1234 cm^−1^ peak is present, these two bands correspond to the γ phase. If both 1275 cm^−1^ and 1234 cm^−1^ peaks appear together, the 840 cm^−1^ and 510 cm^−1^ bands are considered indicative of both β and γ phases [29].

According to the FTIR spectra in Figure 6, with the omission of 1234 cm^−1^ bands, 510 cm^−1^ and 840 cm^−1^ bands can be considered as the β phase.

**Figure 7:**
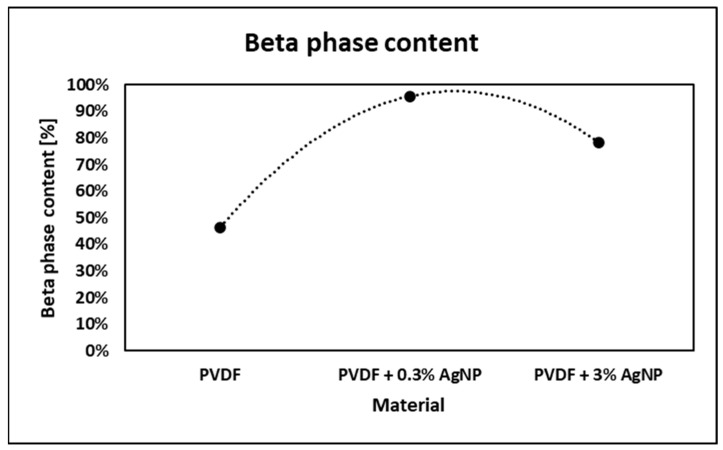
Beta-phase content in different materials.

The relative fraction of the electroactive phase, in this case the β phase, can be quantified using the following formula, according to the Lambert–Beer law [2,29,110]:

(10) $$F_{\beta }=\frac{A_{\beta }}{\left(\frac{K_{\beta }}{K_{\alpha }}\right)A_{\alpha }+A_{\beta }}\times 100$$

where $A_{\alpha }$ and $A_{\beta }$ are the absorbencies at 763 and 840 cm^−1^, respectively, and $K_{\alpha }$ and $K_{\beta }$ are the absorption coefficients at their respective wave numbers, obtained from the literature [2,29,110]. Therefore, the calculated fraction of the β phase in specimens is shown in Figure 7.

Observing obtained FTIR data, it can be concluded that the addition of silver nanoparticles to electrospinning solution promotes PVDF β-phase formation, but only to a certain extent, because further addition of AgNPs led to β-phase content decreasing, as previously confirmed [2,23,102]. Silver nanoparticles have electron-rich surfaces, so their dipole field promotes polymer chain alignment on the AgNPs’ surfaces to form a crystalline polar β phase [111]. In addition. this can facilitate the nucleation of the β phase. However, when the concentration of AgNPs increases further, too many β-phase spherulites are formed which tend to squeeze together, which allows the α phase to be formed in the inter region between these spherulites [111]. Jet instability promotes bead formation, which reduces β-phase content as it prevents adequate fibre stretching [103]. Also, a high concentration of AgNPs can lead to nanoparticle aggregation, which can interfere with the polymer chain alignment necessary for β-phase formation.

### 4.3. Mechanical Testing

**Figure 8:**
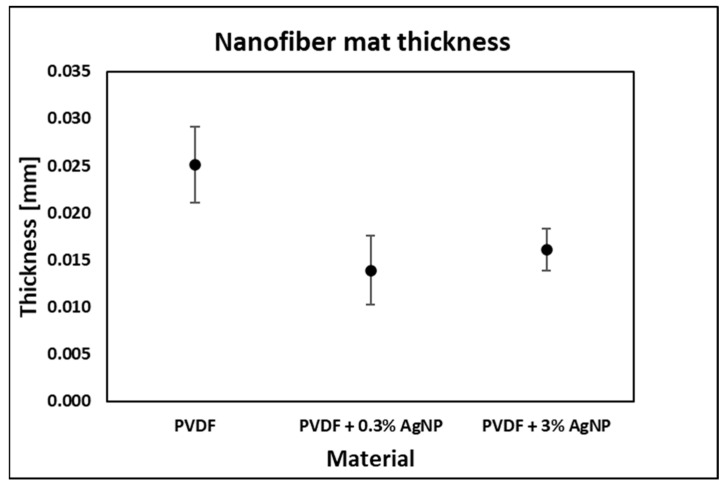
Mean sample thickness values.

Mean sample thickness values, as well as their standard deviations, are presented in Figure 8.

The variation in the thickness of the tested samples is due to the random nature of the electrospinning process. We did not measure the fibre volume variations considering the thickness of the tested samples. However, results from the literature indicate that thicker nanofibers exhibit reduced strain under deformation and, therefore, lower output voltages because the fibre flexibility is decreased, leading to lower mechanical deformation and reduced electrical output [112].

**Figure 9:**
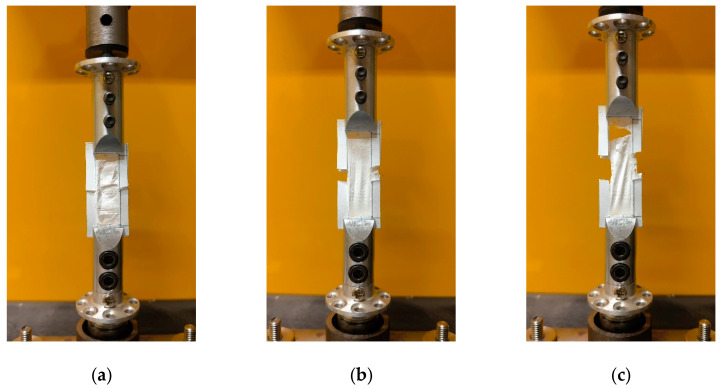
Nanofiber mat tensile testing process: (**a**) beginning; (**b**) middle; and (**c**) at the end of testing.

The nanofiber mat tensile testing process is described in Figure 9, showing the specimen at the beginning of the process inside a paper frame which was cut after placing the sample in the grips, the specimen in the middle of the testing process, and at the end.

**Figure 10:**
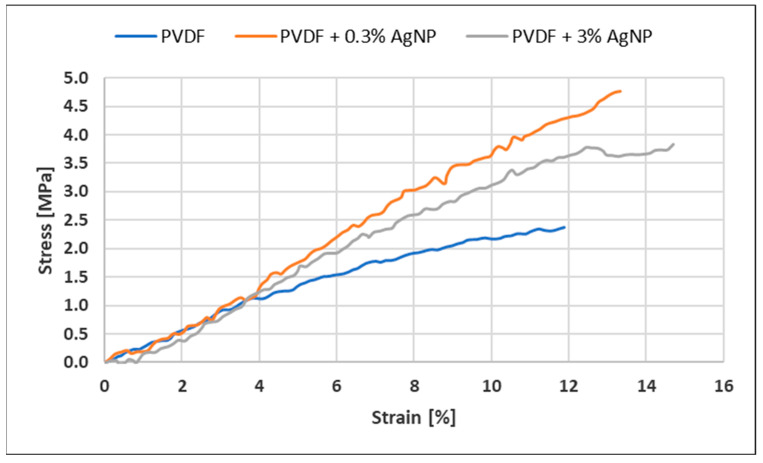
Stress–strain diagrams for different nanofiber mats.

**Figure 11:**
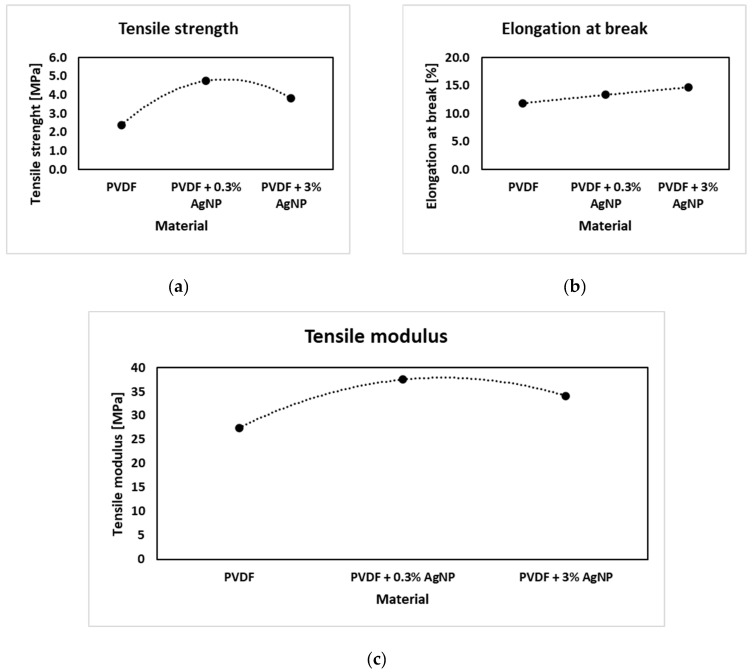
Nanofiber mat tensile testing results: (**a**) tensile strength; (**b**) elongation at break; and (**c**) tensile modulus.

Tensile strength increases with the addition of 0.3% of Ag, as shown in Figure 10 and Figure 11, from around 3 MPa to around 5 MPa for the pure PVDF specimen and PVDF + 0.3% AgNPs, respectively, as similarly reported in [113].

But, further increases in the percentage of AgNPs increase the heterogeneity in the PVDF matrix, which seems to introduce premature flaws in the system at some point which lowers the stress required to fracture [24]. Still, the addition of AgNPs into PVDF leads to a decrease in impurities such as bead formation, as shown in SEM images (Figure 5), which led to improved mechanical properties compared to the pure PVDF specimen. The Ag nanoparticles help hinder crack propagation, even at low concentrations, resulting in increased elongation at break compared to pure PVDF nanofiber mats [24].

### 4.4. Measurements Related to Piezoelectric Outputs

**Figure 12:**
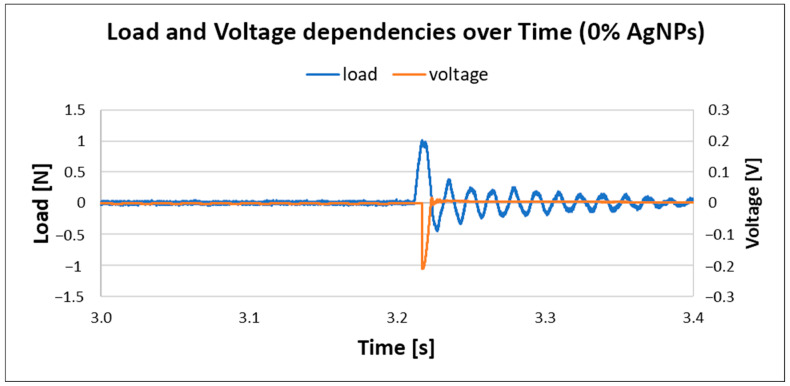
Load and voltage dependency in pure PVDF without AgNPs.

Results for pure PVDF electrospun nanofiber mats are given in Figure 12. The measured load was around 1 N and the generated voltage in this case was around 0.2 V; similar results were reported by references [22,23].

**Figure 13:**
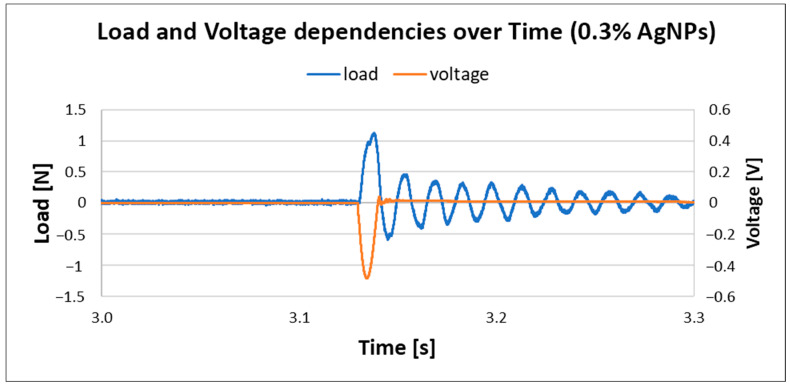
Load and voltage dependency in PVDF with 0.3% AgNPs.

**Figure 14:**
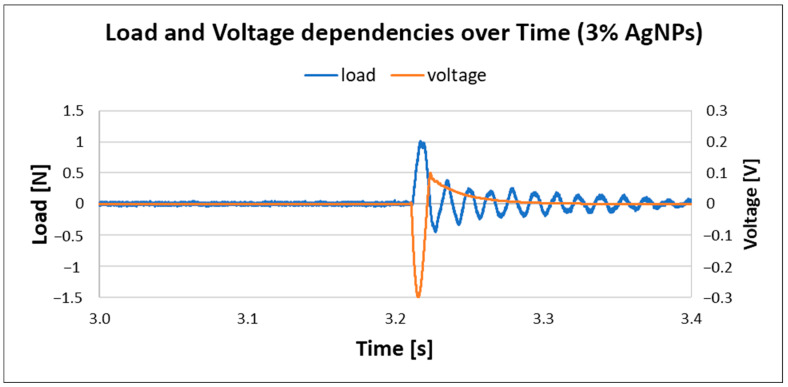
Load and voltage dependency in PVDF with 3% AgNPs.

The results for PVDF nanofiber mats with 0.3% AgNPs are given in Figure 13. The generated load is the same as in the previous case, but with a noted increase in voltage, up to 0.48 V, which suggests that the incorporation of AgNPs into PVDF nanofiber mats increases output voltage; this is in correlation with previously reported work [23]. However, with a further increase in silver nanoparticles in composite material, the voltage output dropped, as shown in Figure 14.

The output voltage corresponds to the beta phase content in the PVDF material, so as the β-phase content increases with the addition of AgNPs, the piezoelectric coefficient increases [102]; hence, the output voltage also increases. However, with higher AgNP concentration, the β-phase content decreases, so the output voltage decreases as well. In addition, it is proven that higher AgNP wt.% leads to a decrease in nanocomposite surface potential, which corresponds to the lower piezoelectric coefficients [19].

Differences in material composition, microstructure and fibre orientations, because of the random nature of the electrospinning process, can cause differ fibre orientations from sample to sample, and can alter mechanical coupling and damping properties. In addition, thickness variations, fibre density and fibre diameters affect how the samples deform or absorb impact, which also affects the attenuation. For instance, the damping behaviour of fibre-reinforced composites is highly dependent on fibre content and orientation [114]. These differences can also lead to different sample conductivity and internal resistance, which also can affect voltage attenuation. Also, if one sample is closer to a resonant frequency of the system, it might exhibit sustained oscillations compared to others because piezoelectric materials generate maximum power at their resonance frequency [115].

### 4.5. Finite Element Analysis

Previously described experimental voltage measurements were used to calculate piezoelectric coefficient $d_{33}$ using Equation (1). This calculated coefficient was implemented in numerical FEA software COMSOL Multiphysics software version 6.3 where electric potential output is simulated.

**Figure 15:**
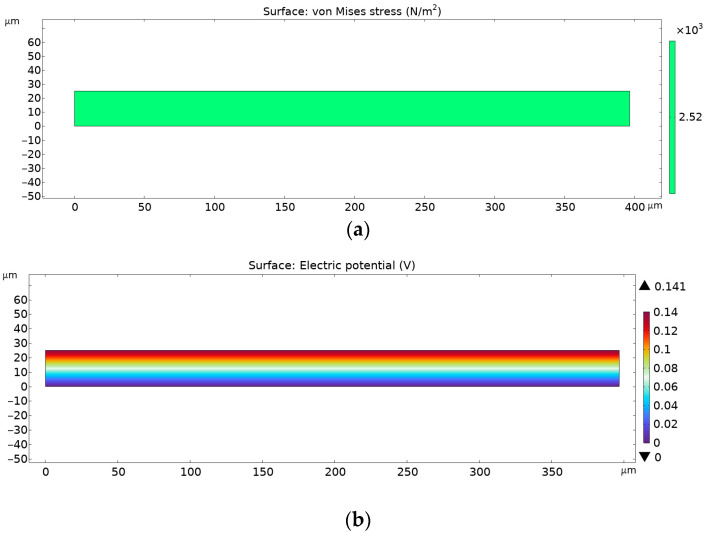
FEA results for pure PVDF nanofibers without (**a**) von Misses stress and (**b**) electric potential.

In Figure 15, numerical results are shown that were obtained through Finite Element Analysis for pure PVDF electrospun nanofiber mats.

**Figure 16:**
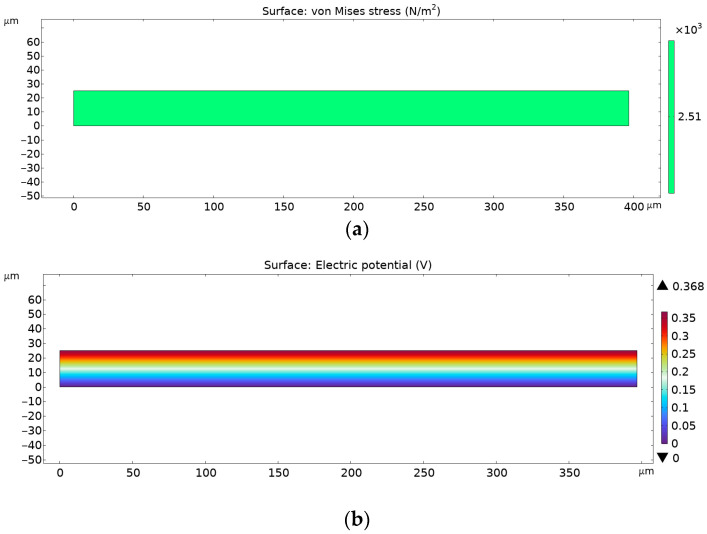
FEA results for PVDF nanofibers with 0.3% AgNPs: (**a**) von Misses stress and (**b**) electric potential.

In Figure 16, numerical results are shown that were obtained through Finite Element Analysis for PVDF electrospun nanofiber mats with 0.3% AgNPs.

**Figure 17:**
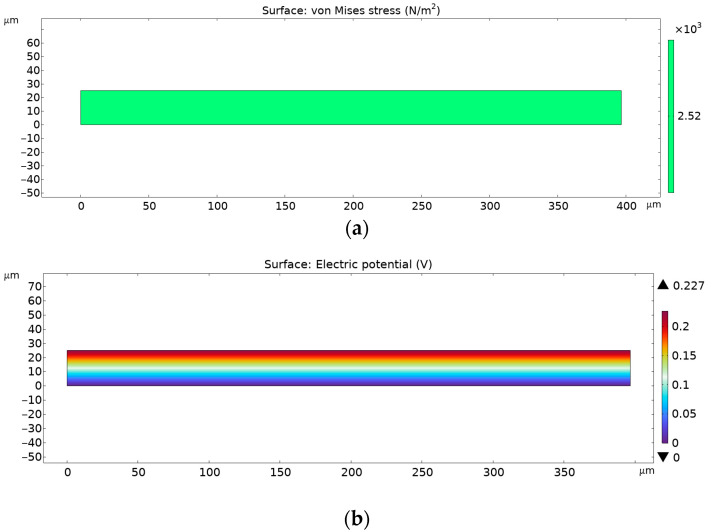
FEA results for PVDF nanofibers with 3% AgNPs: (**a**) von Misses stress and (**b**) electric potential.

Figure 17 denotes FEA results for PVDF + 3% AgNP electrospun nanomats.

As can be observed from the figures above, the von Mises stress is the same for every given case, but the electrical potential is different, due to different piezoelectric coefficient $d_{33}$ values. The pure PVDF nanomats showed a maximum electrical potential of 0.141 V, while those with the addition of 0.3% of AgNPs to PVDF electrical potential exhibited an increase to 0.368 V. However, with further increases in AgNP content in nanomats, to 3%, a decrease in generated voltage was noted, of 0.227 V, which corresponds to the FTIR data and calculated β-phase content. 

**Figure 18:**
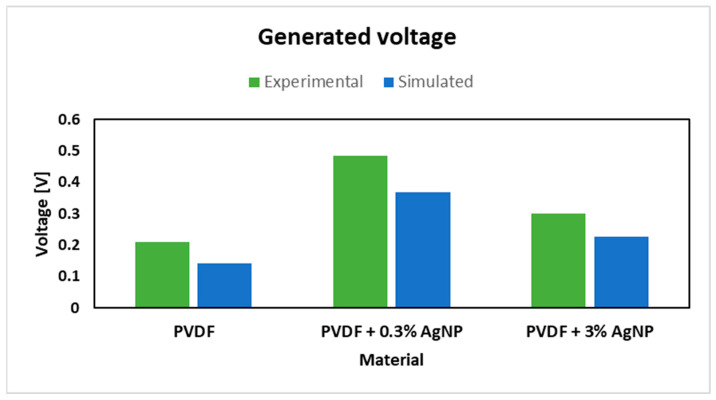
Generated voltage comparison between experimental and simulated data.

Figure 18 describes the comparison of experimental and simulated data for generated voltage where good correlation is found, with minor differences. Pure PVDF and PVDF + 3% AgNP specimens showed greater simulated results compared to experimental specimens, while the PVDF + 0.3% AgNP specimen showed greater experimental output compared to the simulated one, where similar results are reported in the literature, with different filler types and concentrations [32].

We used experimentally determined values of coefficients and material parameters in the 2D FE model and FE analysis, in combination with custom-designed meshing in the FE model with 38 elements (as shown in Figure 2), which showed good correspondence between the experiments and FEM models.

## 5. Conclusions

PVDF with incorporated AgNPs showed enhanced piezoelectricity up to a certain AgNP percentage (0.3%), while, with further increases in AgNP content in the composite, piezoelectricity dropped, compared to the 0.3% composite, but still showed increased piezoelectric response compared to the pure PVDF electrospun nanofiber mats.

SEM analysis proved that the addition of silver nanoparticles to the PVDF electrospun nanofibers provided better homogeneity of the material, removing PVDF beads and increasing overall fibre morphology, while EDS confirmed the presence of AgNPs in the composite due to SEM’s inability to locate nanoparticles because of their size.

FTIR characterization has proven that electrospinning is an effective way to promote the electroactive phase in PVDF nanofiber composites, promoting their piezoelectric properties.

Mechanical tests showed an increase in tensile strength with the addition of 0.3% of AgNPs compared to the specimens without silver particles, while a further increase in AgNPs, to 3%, showed lower tensile strength compared to PVDF with 0.3% AgNPs, probably due to the increase in heterogeneity and the inhibition of premature flaws within the material.

A custom-built measurement setup allows one to tailor and adapt the fixture to specific experimental needs but has disadvantages due to signal instability during tests, so extensive preparations are needed in order to achieve measurement repeatability and reliability. These measurements confirmed that PVDF nanofibers exhibit piezoelectric response, and that the incorporation of AgNPs promotes voltage generation over the same amount of load, which is in agreement with the increase in the electroactive phase in PVDF.

Experimentally obtained results were imported and implemented in numerical modelling software, where FEM analysis showed good agreement with experimental results, thus pointing out that computational models can be used for further virtual experiments focusing on the development of this material for specific applications aiming at better tailoring of the composite piezoelectric outputs.

## Figures and Tables

**Table 1:** Summary of advantages and limitations of different modelling approaches.

Model Type	Advantages	Limitations
Micromechanics Models	- Estimate effective properties based on constituent phases and connectivity types (0-3, 1-3, 3-3). - Simple and efficient for initial approximations.	- Averaging approach may not capture localized behaviours. - Cannot model property fluctuations at small scales, such as phase interfaces.
Models Based on Eshelby’s Equations	- Provide detailed predictions of inclusion effects on overall composite properties. - Suitable for modelling different volume fractions and shape effects.	- Limited by assumptions about phase interactions. - Accuracy depends on inclusion shape and volume fraction approximations.
Dilute Approximation Models	- Simple and computationally efficient. - Works well for low volume fractions of inclusions (assumes weak interactions). - Provides explicit expressions for electroelastic moduli.	- Assumes that inclusions do not interact, making it unreliable for higher volume fractions. - Fails to account for stress and strain interactions between inclusions.
Mori–Tanaka-Based Models	- More accurate than the dilute approximation for moderate inclusion concentrations. - Provides explicit estimates for effective electroelastic moduli. - Works well for aligned inclusions in a dominant matrix phase.	- Assumes uniform stress/strain fields in inclusions, which may not always be realistic. - Assumes that the matrix phase dominates the response, making it less reliable for composites with a high inclusion fraction. - Does not always predict transverse moduli accurately.
Extended Mori–Tanaka Models (e.g., Mori–Tanaka–Eshelby)	- Incorporates Eshelby’s solution for a more precise estimation of inclusion effects.	- Limited in capturing strong interactions between phases.
Self-Consistent Models	- Suitable for composites with moderate inclusion volume fractions. - Captures some level of phase interaction. - Incorporates interaction effects among inclusions. - Provides more realistic predictions compared to the dilute approximation.	- More complex and computationally intensive than simpler models. - Results in an implicit nonlinear algebraic matrix equation for electroelastic moduli, requiring numerical solutions. - Becomes inaccurate when there is a large contrast in the properties of the matrix and inclusions.
Extended Rule of Mixture Models	- Offers a more refined approach to account for phase interactions. - Bridges the gap between simple rule of mixtures and micromechanics models.	- Relies on approximations and assumptions about phase behaviour.
Models Based on Asymptotic Homogenization (or Periodic Homogenization)	- Can capture periodic microstructure effects. - More accurate for composites with ordered phase distribution.	- Computationally demanding. - Requires periodicity assumption, limiting applicability to random structures.
Differential Scheme	- Capable of modelling a wide range of inclusion volume fractions. - More accurate than Mori–Tanaka for high inclusion concentrations. - Accounts for gradual changes in material properties as more inclusions are added.	- Computationally demanding due to the need to solve 81 coupled nonlinear differential equations. - Requires iterative numerical integration.

**Table 2:** The solution and electrospinning parameters [2].

Solution		Electrospinning Parameters				
PVDF Concentration [%]	Solvent [*v*/*v*%]	Voltage [kV]	Needle [Gauge]	Flow Rate [mL/h]	Tip to Collector Distance [cm]	Temperature, Humidity
21%	75% DMF:25% Ac	30	18	0.5	15	30 °C, 45%

## Data Availability

The original contributions presented in the study are included in the article, further inquiries can be directed to the corresponding author.
